# 1762. Clinical application of handheld ultrasound devices during the 2022 Sudan Virus disease Ebola outbreak in Uganda

**DOI:** 10.1093/ofid/ofad500.1593

**Published:** 2023-11-27

**Authors:** Mubaraka Kayiira, Abdullah Wailagala, Jacqueline Nalikkla, Stacy M Kemigisha, Stephen Okello, Isabella H Migisha, Brenda Muhindo, Faith Katusabe, Moses Asiimwe, Kiconco Kyomuhendo, Rosette Kobugabo, Hannah Kibuuka, Peter Waitt, Mohammed Lamorde, Danielle Clark, Paul W Blair

**Affiliations:** Infectious Diseases Institute, Kampala, Kampala, Kampala, Uganda; Infectious Diseases Institute, Kampala, Kampala, Uganda; Makerere University Walter Reed Project, Kampala, Kampala, Uganda; Infectious Diseases Institute, Makerere University, Kampala, Kampala, Uganda; Makerere University Walter Reed Project, Kampala, Kampala, Uganda; Infectious Diseases Institute, Makerere University, Kampala, Kampala, Uganda; Makerere University Walter Reed Project, Kampala, Kampala, Uganda; Infectious Diseases Institute, Makerere University, Kampala, Kampala, Uganda; Makerere University Walter Reed Project, Kampala, Kampala, Uganda; Infectious Diseases Institute, Makerere University, Kampala, Kampala, Uganda; Infectious Diseases Institute, Makerere University, Kampala, Kampala, Uganda; Makerere University Walter Reed Project, Kampala, Kampala, Uganda; Infectious Diseases Institute, Kampala, Kampala, Uganda; Infectious Diseases Institute, Kampala, Kampala, Uganda; The Henry M. Jackson Foundation for the Advancement of Military Medicine, Inc., Bethesda, MD, Bethesda, Maryland; Henry M Jackson Foundation for the Advancement of Military Medicine, Inc, Bethesda, Maryland

## Abstract

**Background:**

The fluid status and causes of respiratory failure in patients with Ebola disease can be challenging to determine due to concomitant severe diarrhea, vascular leakage, and multiorgan failure. Point-of-care ultrasound (POCUS) could guide fluid resuscitation for patients in resource-limited settings. We present the clinical application of POCUS in the 2022 Sudan Virus disease Ebola outbreak in Uganda.

Figure.

**Figure:**
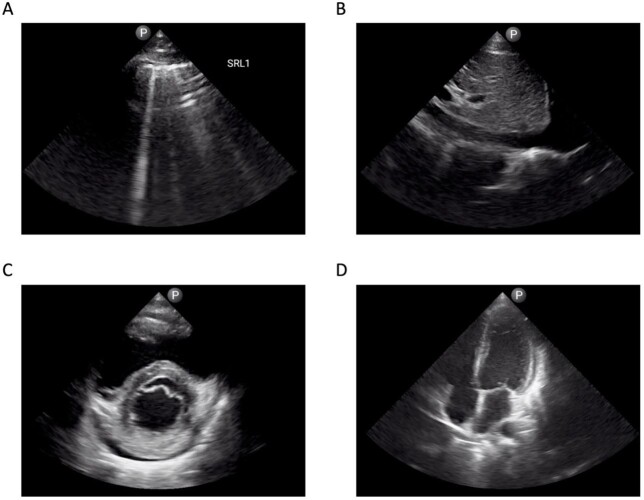

Ultrasound findings from patient 2. 2A: Discrete B lines observed with an irregular pleural line; 2B: Inferior vena cava collapsibility <50% suggestive of adequate fluid resuscitation; 2C: parasternal short cardiac view with small pericardial effusion identified; 2D: apical cardiac view with small pericardial effusion redemonstrated.

**Methods:**

A clinical research team in Fort Portal, Uganda was trained in advanced Infection Prevention and Control (IPC) to care for patients with Ebola disease and in POCUS for septic shock. The clinical team learned a standardized lung and cardiac critical care ultrasound approach to evaluate patients in septic shock. The scanning procedure was used during the care of 6 patients with confirmed Sudan virus disease within 2 weeks of outbreak declaration to make critical patient care decisions like fluid management. Chest X-ray services and mechanical ventilation were not accessible. A handheld Philips Lumify device was kept in the biocontainment area and was used in full personal protective equipment. Scanning procedures included 12-zone lung ultrasound and 4-view cardiac ultrasound scans to determine causes of respiratory disease and evaluate fluid status.

**Results:**

Among 6 patients (age range 27 to 58 years; 1/6 female), 5/6 were in their third week of illness, and 1/6 was in his second week of illness. Lung ultrasound abnormalities included discrete B-lines (5/6 patients), confluent B-lines (1/6), irregular pleural lines (3/6 patients), and subpleural consolidations (1/6 patients). Two participants in respiratory distress had cardiac POCUS. Both had hyperdynamic left ventricular function and one had a small pericardial effusion (Figure). One patient had inferior vena cava (IVC) collapsibility < 50% (1.5 cm diameter), **a**nd another with a >50% IVC collapsibility (1.3 cm diameter). These findings contributed to a decision to continue fluid resuscitation.

**Conclusion:**

Portable handheld ultrasound devices identified previously undescribed ultrasound features of Sudan virus disease. POCUS helped determine causes of respiratory failure and guided fluid resuscitation in a biocontainment ward without access to mechanical ventilation.

**Disclosures:**

**All Authors**: No reported disclosures

